# The assessment of sarcopenia and the frailty phenotype in the outpatient care of older people: implementation and typical values obtained from the Newcastle SarcScreen project

**DOI:** 10.1007/s41999-022-00641-5

**Published:** 2022-04-09

**Authors:** R. M. Dodds, P. Heslop, J. Jaffar, K. Davies, J. M. Noble, F. E. Shaw, M. D. Witham, A. A. Sayer

**Affiliations:** 1 AGE Research Group, Newcastle University Institute for Translational and Clinical Research, Newcastle, UK; 2 NIHR Newcastle Biomedical Research Centre, Newcastle University and Newcastle Upon Tyne NHS Foundation Trust, 3rd Floor Biomedical Research Building, Campus for Ageing and Vitality, Newcastle, NE4 5PL UK; 3 Department of Older People’s Medicine, Newcastle Upon Tyne Hospitals NHS Foundation Trust, Newcastle, UK; 4 School of Design, Northumbria University, Newcastle, UK

**Keywords:** Sarcopenia, Frailty, Grip strength, Walking speed, Implementation, Usual care

## Abstract

**Purpose:**

Sarcopenia and the frailty phenotype both indicate older adults at risk of adverse health outcomes and yet are not widely assessed in practice. We developed the Newcastle SarcScreen to enable assessment of these two ageing syndromes during clinical care. In the setting of our Older People’s Medicine Day Unit, our aims were to describe the implementation of the SarcScreen and to examine the typical values obtained.

**Methods:**

The SarcScreen comprised height, weight, questions (three on the Fried frailty phenotype and five on the SARC-F questionnaire), grip strength and gait speed. We analysed data from 552 patients completing the SarcScreen. We expressed grip strength as *Z*-scores (number of standard deviations above the mean expected for a patient’s age and sex).

**Results:**

It was possible to implement the SarcScreen. In 552 patients (65.9% females) with mean age 80.1 (7.7) years, grip strength was feasible in 98.2% and gait speed in 82.1%. Gait speed was typically not assessed due to mobility impairment. Most patients had weak grip strength (present in 83.8%), slow gait speed (88.8%) and the frailty phenotype (66.2%). We found a high prevalence of probable sarcopenia and the frailty phenotype across all age groups studied. This was reflected by low grip strength *Z*-scores, especially at younger ages: those aged 60–69 had grip strength 2.7 standard deviations (95% CI 2.5–2.9) below that expected.

**Conclusion:**

It is possible to implement an assessment of sarcopenia and the frailty phenotype as part of the routine outpatient care of older people.

**Supplementary Information:**

The online version contains supplementary material available at 10.1007/s41999-022-00641-5.

## Key summary points

**Aim:**

Is it possible to implement the Newcastle SarcScreen, an assessment of sarcopenia and physical frailty, as part of the outpatient care of older people?

**Findings:**

Grip strength measurement was possible in 98.2% and gait speed in 82.1%, with the latter typically not measured due to mobility impairment. We found a high prevalence of probable sarcopenia and the frailty phenotype across all age groups studied.

**Message:**

We successfully implemented the Newcastle SarcScreen. The proforma is available to download as part of this article.

**Supplementary Information:**

The online version contains supplementary material available at 10.1007/s41999-022-00641-5.

## Introduction

The presence of sarcopenia and frailty has been extensively shown to be important in the current and future health of older people [1, 2]. In 2018 the European Working Group on Sarcopenia in Older People 2 (EWGSOP2) consensus defined sarcopenia as a progressive and generalised skeletal muscle disorder, and made recommendations for its assessment in clinical practice [3]. These included the use of the SARC-F questionnaire [4] to identify those at risk of sarcopenia, and the assessment of muscle strength using grip strength [5] and/or the chair stand test [6], with weakness from either being a basis on which to assess potential causes of sarcopenia and start intervention. In the EWGSOP2 consensus, confirmation of the diagnosis of sarcopenia can then made by measurement of muscle mass if available, and severity of sarcopenia assessed using tools such as usual gait speed.

Frailty describes a state of diminished reserve across multiple physiological systems, such that a minor stressor can lead to a marked deterioration in health [2]. A range of approaches have been used to characterise frailty including the frailty phenotype developed by Fried et al. [7]. The frailty phenotype incorporates grip strength and gait speed, as well as questions regarding weight loss, physical inactivity and exhaustion. Hence common to both sarcopenia and the frailty phenotype are the physical function measures of grip strength and gait speed. There is some evidence that sarcopenia and the frailty phenotype can be implemented in routine clinical care [8] but to date this has not been widespread [9].

The assessment of sarcopenia and the frailty phenotype in clinical care is important for several reasons. As above, it identifies patients at risk of poor health outcomes, as well as those who stand to benefit from current treatments for low muscle strength including progressive resistance exercise training and increased dietary protein [10, 11]. The diagnosis of sarcopenia or frailty is also important to aid in the identification of patients who may be eligible for the growing number of trials in patients with these conditions [12]. Finally, most cut-points for measures of physical function are based on normative data from large community-dwelling studies of older people [13, 14] and it is important to establish if such cut-points are helpful to identify older people in clinical care who are at greatest risk of adverse health outcomes.

Our Older People’s Medicine Day Unit sees older people referred from primary care, many of whom have complex multimorbidity as well as impairment of physical function. Patients receive a comprehensive geriatric assessment (CGA) delivered by a multidisciplinary team (MDT) [15]. In 2018 we developed and incorporated the Newcastle SarcScreen, an assessment of sarcopenia and the frailty phenotype, as part of the CGA process we deliver. The aims of this study were (i) to describe the implementation of sarcopenia and the frailty phenotype into the routine care of patients attending our Day Unit, and (ii) to compare the SarcScreen values obtained from a large sample of our patients to recommended cut-points and normative data.

## Methods

### Clinical setting and patients

Our Older People’s Medicine Day Unit is one of the specialist services provided by Newcastle Hospitals in northeast England. The SarcScreen proforma was completed by the Day Unit nurses as part of the routine assessments they carried out on all new patients. We examined results from SarcScreen proformas completed between June 2018 and March 2020, at which time in-person clinical reviews were temporarily suspended due to the COVID-19 lockdown. The data were collected on a spreadsheet and stored within the Trust network with approval from the local Caldicott guardian. As the analysis used data already collected as part of routine clinical care, with no new patient contact or additional data collection, the project did not require evaluation by a research ethics committee. Data were anonymized within the trust before export for analysis.

### Implementation of the Newcastle SarcScreen

The Day Unit nurses assessed the components of SarcScreen, following training sessions delivered by an experienced researcher (KD) when the SarcScreen was introduced. A standard operating procedure document was also provided. We used feedback from colleagues in the Day Unit and observation of the tool being used to refine the proforma, as described in the results. Height (cm) and weight (kg) were either measured or reported by the patient. Three questions covered the components of the Fried frailty phenotype as follows: (i) unintentional weight loss of more than 10 lb in the last year, (ii) frequency of physical activity requiring a low or moderate level of energy (with less than once a week considered to be low physical activity), and (iii) number of days in the past week where everything was an effort for a patient or they could not get going (with 3 or more days considered to represent exhaustion) [7]. Further questions addressed the five components of the SARC-F questionnaire, comprising difficulty in (i) lifting and carrying 10 pounds, (ii) walking across a room, (iii) transferring from a chair or bed, and (iv) climbing a flight of ten stairs, with all scored zero for no difficulty, one for some and two for those with a lot of difficulty/unable to perform the activity in question. A fifth question asked about the number of falls in the past year, scored zero for none, one for 1–3 times and two for four or more falls. A total score of four or more indicates a high risk of sarcopenia being present [4]. The SARC-F questions were not included in the initial version of the proforma (meaning that for the first 37 patients these questions were not attempted).

Grip strength was measured using a Jamar hydraulic dynamometer in the seated position with the patient’s forearm rested on the arm of a chair, with the weight of dynamometer supported by the assessor [5]. In an initial version of the proforma we only attempted one trial in each hand (*n* = 37), but thereafter changed to two trials in each hand. We took the maximum of the available trials. Males with a strength below 27 kg and females below 16 kg, the cut-points for probable sarcopenia endorsed by the EWGSOP2 guideline, were considered to have weak grip strength [3]. Gait speed was attempted in patients who could usually walk without the assistance of another person, and where the nurse carrying out the assessment considered it was safe to proceed. Patients were timed walking along a marked 3 m course, with the stopwatch started when their foot crossed the first marker and stopped when they crossed the second. A gait speed of 0.8 m/s or below was considered to be slow [14, 16]. There was space to note a reason if it was not possible to complete the grip strength and/or gait speed tests.

The presence of the three frailty questions, weak grip strength, and slow gait speed were used to create a Fried frailty score out of five. We classed patients unable to complete tests due to health reasons as having poor performance for the purpose of the Fried frailty score [17]. Frailty was indicated by a score of three or more, and pre-frailty by a score of one or two. A copy of the proforma is provided in Supplementary Material 1.

### Statistical analysis

We calculated the proportion of patients completing each component of the SarcScreen. We tested if there were age or sex differences in the SarcScreen components. *T* test was used for comparison of mean values, and a chi-square test for proportions. We compared our grip strength values to published British normative data [13], by expressing each patient’s value as a *Z*-score. Each *Z*-score is calculated as the patient’s grip strength value less the mean expected for their age and sex, divided by the grip strength standard deviation (SD) for their age and sex. As such *Z*-scores have no units (kg/kg). *Z*-scores of + 1 and − 1 indicate grip strength values one standard deviation above and below, respectively, that expected for age and sex. We divided the sample into age groups by decade (60–69, 70–79 and so on) and calculated the mean and 95% confidence interval for the *Z*-score in each age group (having first checked by inspection of the data that the *Z*-scores were normally distributed). We performed all analyses using Stata version 14.0 [18].

## Results

### Implementation of the SarcScreen

We did not experience any adverse events related to the use of the SarcScreen. The assessment was readily incorporated into the range of other measures such as testing of cognition during a CGA. Feedback from colleagues in the MDT highlighted how measures such as grip strength gave an easy-to-interpret assessment of a person’s physical function, helpful for situations such as in discussion at an MDT meeting including as a means for identifying patients who might have particular benefit from Physiotherapy input. We explored the incorporation of multi-segmental bioimpedance to assess muscle mass, although did not proceed due to concerns that the step-up onto the device presented a falls risk. We developed a standard operating procedure document to accompany the original SarcScreen proforma. We found that in practice this was not always easy for team members to locate, and so modified the form to include a summary of the instructions for the grip strength and gait speed assessment. The SarcScreen proforma is provided in Supplementary Material 1.

It was possible to complete the body size and questionnaire measures in almost all patients: height and weight in 99.3%, questionnaire components of the Fried frailty phenotype in 96.0%, and the SARC-F questionnaire in 94.2% of those attempted. It was possible to measure grip strength in 98.2% of patients, with a further 1.1% unable to attempt due to health reasons (in all cases due to pain in the wrist or hand such as from arthritis) and no reason recorded in 0.7% of patients. It was possible to measure gait speed in 82.1% of patients (of whom 38.4% used a walking aid). Gait speed was not completed in 8.3% of patients due to reduced mobility, 1.3% due to breathlessness and in a further 8.3% no reason was recorded. Non-completion of gait speed was more common in those with a SARC-F score of eight or more, and those reporting low physical activity or exhaustion. A Fried frailty phenotype score could be assigned in 87.9% of patients.

### Examination of values obtained from the SarcScreen

**Fig. 1:**
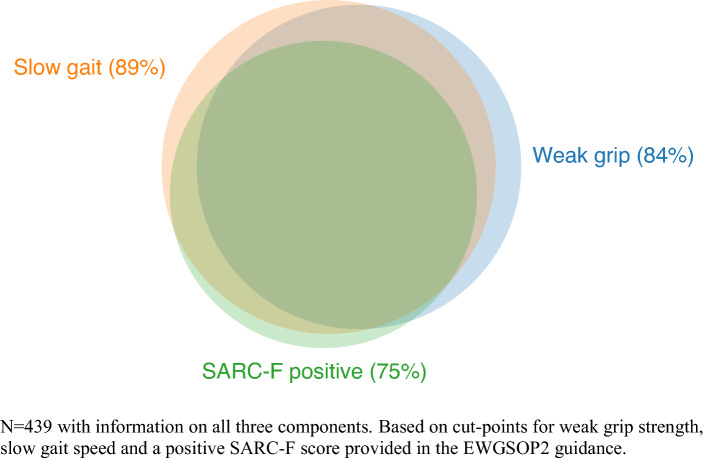
Venn diagram showing overlap of sarcopenia components. *n* = 439 with information on all three components. Based on cut-points for weak grip strength, slow gait speed, and a positive SARC-F score provided in the EWGSOP2 guidance

We examined results from 552 patients (65.9% female) with mean age 80.1 (7.7) years assessed using the SarcScreen. There were no marked sex differences in the values obtained, as shown in Table 1. The mean BMI of participants was in the overweight range at 27.4 (6.93) kg/m^2^. A SARC-F score of 4 or more (present in 75.2%), weak grip strength (present in 83.8%), and slow gait speed (present in 88.8%) as per the EWGSOP2 cut-points were all common. Approximately one-quarter (24.4%) were below cut-points for two of the three measures, and approximately two-thirds (63.3%) were below the cut-points for all three. This is shown in the form of a Venn diagram in Fig. 1. The questionnaire components of the Fried frailty phenotype were also common, especially low physical activity (present in 38.0%) and exhaustion (present in 54.9%). This combined with the grip strength and gait speed values meant that Fried frailty was present in 66.2% of the sample.

**Table 1:** Summary values from Newcastle SarcScreen, by sex

	*n*	All (max *n* = 552)	Males (max *n* = 188)	Females (max *n* = 364)	*P *value for sex difference*
Age (years)	552	80.1 (7.7)	80.1 (7.7)	80.1 (7.7)	0.9
BMI (kg/m^2^)	548	27.4 (6.93)	27.2 (5.0)	27.5 (6.9)	0.6
SARC-F	485				0.09
0–3 [*n* (%)]		122 (25.2)	51 (31.3)	71 (22.1)	
4–7 [*n* (%)]		234 (48.3)	72 (44.2)	162 (50.3)	
8–10 [*n* (%)]		129 (26.6)	40 (24.5)	89 (27.6)	
Grip strength (kg)	542	Not reported	18.9 (7.1)	10.9 (5.7)	Not tested
Weak grip strength [*n* (%)]^†^	548	459 (83.8%)	161 (86.1%)	298 (82.6%)	0.3
Gait speed (m/s)	453	0.5 (0.2)	0.6 (0.2)	0.5 (0.2)	0.048
Slow gait speed [*n* (%)]^†^	506	454 (89.7%)	149 (87.1%)	305 (91.0%)	0.2
Unintentional weight loss [n (%)]	542	142 (26.2%)	52 (28.1)	90 (25.2)	0.5
Low physical activity [n (%)]	547	208 (38.0%)	73 (39.5%)	135 (37.3%)	0.6
Exhaustion [*n* (%)]	545	299 (54.9%)	96 (51.6%)	203 (56.6%)	0.3
Fried phenotype	485				0.2
0 (Non-frail)		12 (2.5%)	2 (1.2%)	10 (3.1%)	
1–2 (Pre-frail)		152 (31.3%)	59 (35.8%)	93 (29.1%)	
3+ (Frail)		321 (66.2%)	104 (63.0%)	217 (67.8%)	

All mean (SD) unless otherwise stated

*From a *t* test for comparison of mean values, and a chi-square test for proportions

^†^Includes those unable to perform the test due to health reasons

**Fig. 2:**
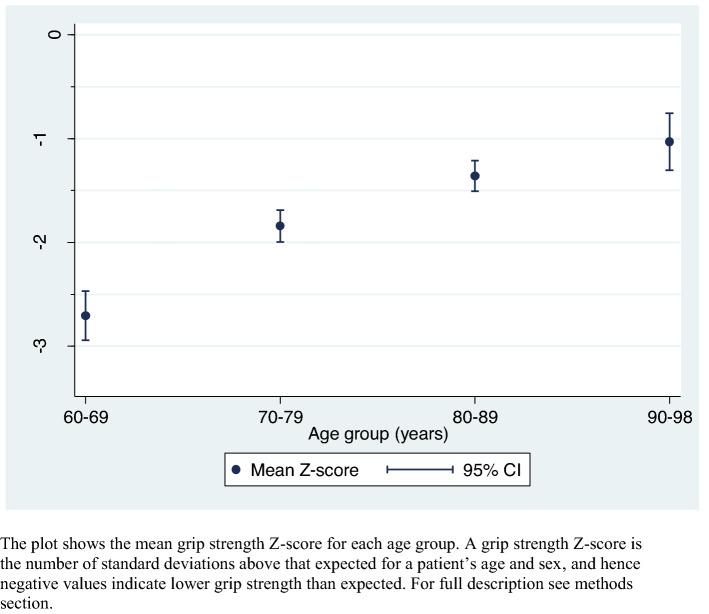
Grip strength values expressed relative to British normative data, by age group. The plot shows the mean grip strength *Z*-score for each age group. A grip strength *Z*-score is the number of standard deviations above that expected for a patient’s age and sex, and hence negative values indicate lower grip strength than expected. For full description see “Methods” section

We did not find evidence of an increase in the prevalence of a positive SARC-F score, weak grip strength, slow gait speed or Fried frailty with age, but rather we observed similar values across the different age groups. For example, the prevalence of a positive SARC-F score was 74.1% below age 81 (the median age of the sample) and 75.6% at age 81 and above. The Z-scores for grip strength by age group showed that all age groups had mean grip strength values below those expected for their age and sex based on existing British normative data. This was especially the case in younger patients, as shown in Fig. 2. For example, patients aged 60–69 had a mean grip strength 2.7 standard deviations (95% CI 2.5, 2.9) below that expected, compared to patients aged 90–98 where the mean was 1.0 standard deviations (95% CI 0.8, 1.3) lower.

## Discussion

We successfully implemented an assessment of sarcopenia and the frailty phenotype as part of the routine outpatient care of older people attending our Older People’s Medicine Day Unit. We have shown that completion of relevant questionnaire measures and of grip strength was possible in nearly all patients. Measurement of gait speed was not completed in almost one-fifth of patients, although this was associated with a raised SARC-F score and hence is likely to be a marker of poor mobility. There was a high prevalence of poor performance such as weak grip strength and this was similar in both younger and older age groups.

The CGA process that we use in the Day Unit already included several questionnaire measures and so we anticipated that the SARC-F and Fried frailty questions would be possible to implement. We also found that grip strength measurement was possible, with only a small proportion of patients (approximately 1%) unable to complete the assessment. This was less than in a study of the implementation of grip strength in acute hospital wards for older people, where a range of health issues including acute illness and severe confusion led to around one-fifth of patients being unable to complete grip strength [8]. In a similar manner, a research study conducted with older patients on acute medicine wards found that gait speed was not possible in a much higher proportion of patients than seen in our Day Unit (66% compared to 18%) [19].

In terms of the typical values in our patient group, two findings stood out. First, we observed a high proportion of patients who fell below the relevant cut-points in the EWGSOP2 definition, or who met the criteria for the Fried frailty phenotype, or both. For example, 84% of patients fell below the EWGSOP2 cut-points for grip strength. This contrasts with a lower proportion of weak grip strength seen in a research study of sarcopenia prevalence in another Day Unit setting of 49% [20]. A range of factors are likely to explain this difference, but one possibility is that older people with low strength may be less likely to be recruited into research. The high prevalence of weak grip strength (and hence probable sarcopenia) highlights the importance of having access to resistance exercise interventions for this patient group [10]. Another benefit of identifying probable sarcopenia and/or the frailty phenotype in patients is that it may facilitate recruitment to the growing number of research studies including trials in this area [12].

Second, we observed similar grip strength values in young-old and older patients, in contrast to findings from normative data from the general population where mean grip strength tends to decline from mid-life onwards [13]. This was highlighted by our finding of a significantly lower mean *Z*-score among the young-old compared to the older age groups, and may reflect the nature of patients seen in our Day Unit, where functional impairment (including mobility impairment) is a key reason for referral regardless of age. These two findings highlight the importance of analysing data collected during routine care, which may in turn suggest lower cut-points to identify those most at risk. Related to this, recent analyses performed by the Sarcopenia Definitions and Outcomes Consortium suggest that a cut-point for gait speed of 0.6 m/s performs better than 0.8 m/s in identifying individuals with mobility limitation [21].

Limitations of the present study include that we did not assess chair stand test performance [6] or muscle mass. These are areas for future work in the development of the SarcScreen. The results of the paper proforma were also not directly stored in our hospital’s electronic healthcare record. We have recently implemented a version of the SarcScreen with direct storage of results in the electronic healthcare record and in the future this should enable further analyses including associations with outcomes [22]. We used information from saved copies of the SarcScreen proforma and it is possible that some patients attending did not have a form completed, although to our knowledge this was uncommon. A strength is that we collected and analysed a large sample of routine data relevant to the outpatient care of older people. This included the reasons for non-completion of physical performance tests, with health-related reasons having previously been shown to be associated with adverse health outcomes including reduced survival times [17].

In conclusion, it is possible to implement an assessment of sarcopenia and the frailty phenotype as part of the routine care of older people attending an Older People’s Medicine Day Unit. The Newcastle SarcScreen can be used to identify these ageing syndromes which are amenable to treatments such as progressive resistance exercise training, and are also the focus of a growing number of clinical trials. Most patients fell below cut-points proposed in the EWGSOP2 definition, and hence an area for future work is to examine these and other cut-points in the clinical prediction of key health outcomes.

## Supplementary Information

Below is the link to the electronic supplementary material.

Supplementary file1 (PDF 54 kb)
