# Supplementary material for: The assessment of sarcopenia and the frailty phenotype in the outpatient care of older people: implementation and typical values obtained from the Newcastle SarcScreen project
Source: Eur Geriatr Med. 2022 Apr 9;13(4):763–9. doi: 10.1007/s41999-022-00641-5 (PMC8995690; doi:10.1007/s41999-022-00641-5)
Supplement: Supplementary file 1 — Supplementary file1 (PDF 54 kb) [file 41999_2022_641_MOESM1_ESM.pdf]

The Newcastle upon Tyne Hospitals NHS Foundation Trust

Newcastle SarcScreen

Newcastle University

Attach patient label

Must include NHS number

Date: ☐☐ / ☐☐ / ☐☐

Location: ☐

☐ Day Unit ☐ Clinic

If Clinic, which? ☐

1. Height: ☐☐☐ cm ☐ Height measured ☐ Patient reported

2. Weight: ☐☐☐ . ☐ kg ☐ Weight measured ☐ Patient reported

3. BMI: ☐☐ . ☐ kg/m^2

4. Questions to ask patient / relative / carer:

a. In the past year have you lost more than 10lb in weight unintentionally (i.e. not due to dieting or exercise)?

☐ Yes ☐ No

b. How often do you engage in activities that require a low or moderate level of energy such as light housework (vacuuming, ironing, making the bed), going for a walk, gardening or cleaning the car?

☐ Hardly ever ☐ 1-3 times a month ☐ Once a week ☐ More than once a week

c. On how many days during the past week have you felt that everything you did was an effort or that you could not get going?

☐ None ☐ 1-2 days ☐ 3-4 days ☐ 5-7 days

d. How much difficulty do you have in lifting and carrying 10 pounds (around 5kg or 5 bags of sugar)?

☐ None ☐ Some ☐ A lot or unable

e. How much difficulty do you have in walking across a room?

☐ None ☐ Some ☐ A lot, use aids or unable

f. How much difficulty do you have transferring from a chair or bed?

☐ None ☐ Some ☐ A lot or unable without help

g. How much difficulty do you have climbing a flight of 10 stairs?

☐ None ☐ Some ☐ A lot or unable

h. How many times have you fallen in the past year?

☐ None ☐ 1-3 times ☐ 4 or more times

5. Hand grip strength (please see protocol sheet for full details):

- **Do not continue with the test if the patient finds it uncomfortable**
- Demonstrate how to hold the dynamometer and show how the dial registers when you squeeze as tightly as possible
- Start with the right side
- Ask the patient to rest their forearm on the arm of the chair with their wrist just over the end of the arm of the chair and their thumb facing upwards
- Check that the dynamometer feels comfortable in their hand. The position of the handle can be adjusted if necessary for different sized hands
- Support the base of the dynamometer in the palm of your hand, so you take the weight. Inform the patient it will feel as if there is no resistance
- Encourage the patient to squeeze as long and as tightly as possible or until the needle stops rising. Record the grip strength in kg from the outside dial
- Repeat for the left hand, and then repeat for right and left hands (2nd attempt)

1st attempt: Right hand ☐☐ kg Left hand ☐☐ kg

2nd attempt: Right hand ☐☐ kg Left hand ☐☐ kg

If unable to do grip, record why: ☐

---

6. Gait speed (please see protocol sheet for full details):

- **Do not attempt if you are concerned that the patient is not safe to walk**
- Patients who need the assistance of another person to walk do not need to do this test – please record below
- Patients who use walking aids can do the test but please record below if used
- Check that a marked walkway and stopwatch are available
- Ask the patient to stand behind the first marker. Say "I will say ready, set, go. When I say go, walk at your normal comfortable speed until I say stop"
- Say go and start the stop watch when their foot crosses the first marker
- Walk alongside the patient and stop the stopwatch when their foot crosses the second marker (even if the foot is in mid-air). Record the distance walked and the time below
- Thank the patient and ensure they have somewhere to sit comfortably.

1st walk distance ☐ metres Time taken to do walk ☐☐ . ☐ seconds

Some clinics use a second walk test. If done, record below:

2nd walk (if done) ☐ metres Time taken to do walk ☐☐ . ☐ seconds

If used an aid, record which: ☐

If did not do walk, record why: ☐
